# Complement Activation Contributes to Severe Acute Respiratory Syndrome Coronavirus Pathogenesis

**DOI:** 10.1128/mBio.01753-18

**Published:** 2018-10-09

**Authors:** Lisa E. Gralinski, Timothy P. Sheahan, Thomas E. Morrison, Vineet D. Menachery, Kara Jensen, Sarah R. Leist, Alan Whitmore, Mark T. Heise, Ralph S. Baric

**Affiliations:** a Department of Epidemiology, University of North Carolina, Chapel Hill, North Carolina, USA; b Department of Immunology and Microbiology, University of Colorado School of Medicine, Aurora, Colorado, USA; c Department of Microbiology and Immunology, University of Texas Medical Branch, Galveston, Texas, USA; d Department of Genetics, University of North Carolina, Chapel Hill, North Carolina, USA; NIAID, NIH; Centro Nacional de Biotecnologia, CNB-CSIC; St. Jude Children's Research Hospital

**Keywords:** SARS-CoV, animal models, complement, coronavirus, respiratory viruses

## Abstract

Acute respiratory distress syndrome (ARDS) is immune-driven pathologies that are observed in severe cases of severe acute respiratory syndrome coronavirus (SARS-CoV) infection. SARS-CoV emerged in 2002 to 2003 and led to a global outbreak of SARS. As with the outcome of human infection, intranasal infection of C57BL/6J mice with mouse-adapted SARS-CoV results in high-titer virus replication within the lung, induction of inflammatory cytokines and chemokines, and immune cell infiltration within the lung. Using this model, we investigated the role of the complement system during SARS-CoV infection. We observed activation of the complement cascade in the lung as early as day 1 following SARS-CoV infection. To test whether this activation contributed to protective or pathologic outcomes, we utilized mice deficient in C3 (C3^–/–^), the central component of the complement system. Relative to C57BL/6J control mice, SARS-CoV-infected *C3*^–/–^ mice exhibited significantly less weight loss and less respiratory dysfunction despite equivalent viral loads in the lung. Significantly fewer neutrophils and inflammatory monocytes were present in the lungs of *C3*^–/–^ mice than in C56BL/6J controls, and subsequent studies revealed reduced lung pathology and lower cytokine and chemokine levels in both the lungs and the sera of *C3*^–/–^ mice than in controls. These studies identify the complement system as an important host mediator of SARS-CoV-induced disease and suggest that complement activation regulates a systemic proinflammatory response to SARS-CoV infection. Furthermore, these data suggest that SARS-CoV-mediated disease is largely immune driven and that inhibiting complement signaling after SARS-CoV infection might function as an effective immune therapeutic.

## Abstract (precis)

The complement system is a critical part of host defense to many bacterial, viral, and fungal infections. It works alongside pattern recognition receptors to stimulate host defense systems in advance of activation of the adaptive immune response. In this study, we directly test the role of complement in SARS-CoV pathogenesis using a mouse model and show that respiratory disease is significantly reduced in the absence of complement even though viral load is unchanged. Complement-deficient mice have reduced neutrophilia in their lungs and reduced systemic inflammation, consistent with the observation that SARS-CoV pathogenesis is an immune-driven disease. These data suggest that inhibition of complement signaling might be an effective treatment option following coronavirus infection.

## INTRODUCTION

Severe acute respiratory syndrome coronavirus (SARS-CoV) emerged in 2002 and 2003 from coronaviruses circulating in animal markets in China (1). Emergence of this novel virus led to a global outbreak of respiratory disease, with over 8,000 human cases and 10% mortality (2, 3). In 2012, a new, related zoonotic coronavirus was identified in the Middle East, designated Middle East respiratory syndrome coronavirus (MERS-CoV), causing severe respiratory disease with greater than 35% mortality (www.who.int/emergencies/mers-cov/en) (4). Both SARS-CoV and MERS-CoV cause a range of disease from asymptomatic cases to severe acute respiratory distress syndrome (ARDS) and respiratory failure (5). Notably, metagenomics and synthetic virus recovery strategies have since revealed the existence of large pools of preepidemic SARS-like bat coronaviruses which replicate in primary human airway epithelial cells. These viruses are poised for emergence because they both efficiently use human ACE2 entry receptors and resist existing vaccines and immunotherapeutics (6, 7). Due to the ongoing threat and continued emergence of new, highly pathogenic coronaviruses from animal reservoirs, a thorough understanding of the host-virus interactions that drive SARS-CoV pathogenesis will aid the public health response to current and future coronavirus outbreaks (8).

The importance of complement in SARS-CoV pathogenesis is controversial. Previous studies have investigated the role of known polymorphisms in the mannose-binding lectin (*MBL*) and MBL-associated serine protese-2 (*MASP2*) genes in SARS-CoV infection outcome following the 2003 outbreak but with conflicting results. One retrospective analysis showed that people with low or deficient serum MBL levels were more likely to become infected with SARS-CoV (9) than those with high MBL levels, suggesting that MBL and complement activation play a role in protecting the host from infection. However, a second study found no association between *MBL* haplotype and SARS-CoV infection status (10). Additionally, it was shown MBL can bind to the SARS-CoV Spike protein *in vitro* by some groups (11) but not by others (12). Examination of the role of the downstream complement gene *MASP2* found no association between genotype and SARS susceptibility (13). Together, the results leave a general uncertainty about the role of complement in response to SARS-CoV infection.

Despite the existing body of literature, the role of complement in SARS-CoV pathogenesis has never been directly assessed *in vivo*. The complement system is an ancient arm of the innate immune response comprised of multiple proteins whose reactive cascade of cleavage products can coordinate the inflammatory response at the sites of infection and can be directly antimicrobial. Consisting of more than 30 soluble and cell surface-associated proteins, complement is a major component of innate immunity that functions to recognize and eliminate invading pathogens (14). Activation of the complement system occurs through multiple mechanisms that include three well-described pathways, the classical, lectin, and alternative complement activation pathways (15), and results in proteolytic processing of various components of the complement system, including C3, C4, and C5. Proteolytic processing of C3 generates an array of cleavage products that are involved in amplification of complement activity through formation of C3 and C5 convertases, opsonization of pathogens, and attraction and activation of leukocytes of both the innate and adaptive arms of the immune response. Several studies, including a recent study showing that complement blockade results in reduced disease in a MERS-CoV human DPP4 transgenic (*h*DPP4-Tg) mouse model (16), have elucidated protective and pathogenic roles for the complement system following infection by a variety of viral pathogens (17). Furthermore, the complement system has well-described roles in other pulmonary diseases (18), especially after influenza virus and respiratory syncytial virus infection (19–21).

In this study, we assessed the role of the complement system in the pathogenesis of SARS-CoV infection. Building from a systems biology analysis that suggested that complement was modulated during SARS-CoV infection, we confirmed that complement was activated upon SARS-CoV challenge. Mice deficient in C3 (*C3*^–/–^), the central protein of the complement signaling pathway, were protected from SARS-CoV-induced weight loss and had reduced pathology, improved respiratory function, and lower levels of inflammatory cytokines/chemokines in the lung and periphery. Importantly, the kinetics and magnitude of virus replication in *C3*^–/–^ and wild-type mice were the same, showing that complement does not play a role in controlling virus replication. We observed complement deposition in the lungs of SARS-CoV-infected mice, suggesting that complement activation results in immune-mediated damage to the lung. Additionally, serum activation indicates that complement-mediated systemic inflammation may drive the pathogenic response to SARS-CoV infection. Together, the results indicate that complement plays a critical role in SARS-CoV pathogenesis and that inhibition of the complement pathway might be an effective therapeutic to coronavirus-mediated disease.

## RESULTS

### Complement is activated in SARS-CoV MA15-infected mice.

**FIG 1:**
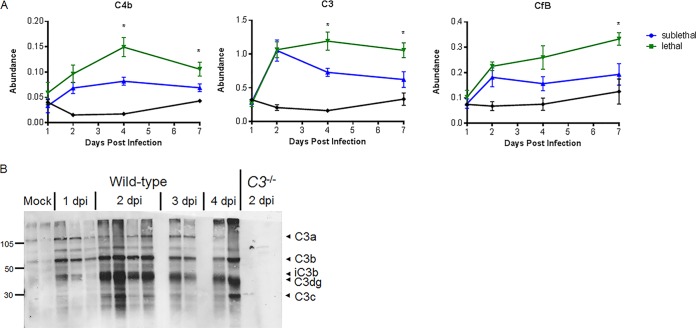
Omics characterization of complement pathway expression and activation. (A) Protein abundance at 1, 2, 4, and 7 days postinfection relative to that in mock-treated samples. Samples were taken from total lung homogenates, and error bars indicate standard errors of the means (SEM). Each point indicates the mean of results for 5 mice at a given time. (B) C3 protein cleavage is observed in the lung by Western blotting as early as 24 h following SARS-CoV MA15 infection of C57BL/6J mice. Numbers at the left are molecular weights (in thousands).

While work by other laboratories has shown that C3-deficient mice are extremely susceptible to both H5N1 and H1N1 influenza virus infection (22), the role of complement in SARS-CoV infection has not yet been evaluated *in vivo*. Using a systems biology-based approach, we identified the complement pathway as a high-priority target for control of SARS-CoV MA15 (the mouse-adapted SARS-CoV) pathogenesis based on weighted gene correlation network analysis (WGCNA) of RNA transcripts in the lungs of mice infected with a lethal versus a sublethal dose of SARS-CoV MA15 (23). Because the complement signaling cascade is activated through proteolytic cleavage events, we also assessed lung proteomics samples for complement protein abundance. C4b, CfB, and C3 all had significantly higher abundances in the lungs of mice infected with a lethal dose of SARS-CoV MA15 than in those of mice infected with a sublethal dose (Fig. 1A). Complement activation is measured by detection of pathway component cleavage products. C3, the master regulator of complement signaling, is cleaved into C3a and C3b following creation of C3 convertase. C3 activation products (C3 fragments C3a, C3b, iC3b, C3dg, and C3c) were detected by Western blotting in lung tissue of SARS-CoV MA15-infected mice, but not in control mice, as early as 1 day postinfection (dpi) (Fig. 1B), confirming that SARS-CoV MA15 infection activates the complement pathway.

### Multiple complement pathways contribute to SARS-CoV MA15-induced pathogenesis.

**FIG 2:**
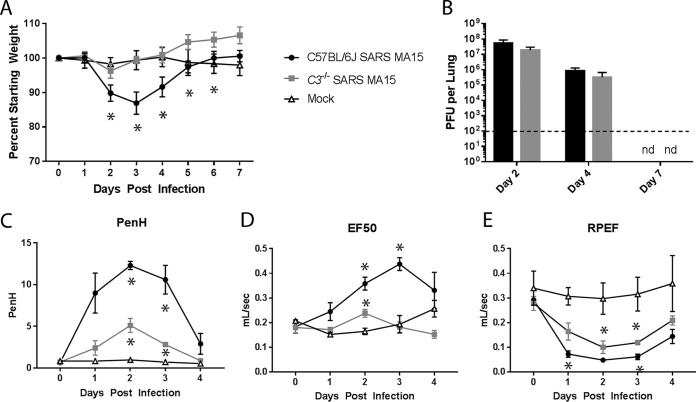
Characterization of C3 knockout mice. (A) Weight loss of SARS-CoV MA15-infected C57BL/6J mice, *C3*^–/–^ mice, or mock-infected mice were measured over time. (B) Lung titers of SARS-CoV MA15-infected C57BL/6J or C3^–/–^ mice at 2, 4, and 7 days postinfection. nd, not determined. (A and B) Six to 8 mice were used through day 4, and 3 to 4 mice were used for days 5 to 7. The respiratory function of SARS-CoV MA15-infected C57BL/6J and *C3*^–/–^ mice and mock-infected mice was measured using a Buxco whole-body plethysmography system for Penh, a measure of calculated airway resistance (C), EF50, midbreath expiratory flow (D), and RPEF, the rate of peak expiratory flow (E). *, *P* < 0.05 between mock-infected mice and a given condition. (C to E) Three mice were used for each infection group, and two mock-infected mice were used.

To test the importance of the complement signaling pathway in SARS-CoV pathogenesis, we infected *C3*^–/–^ mice and C57BL/6J controls with SARS-CoV MA15. Control mice exhibited approximately 15% transient weight loss, with peak weight loss at day 3 postinfection (Fig. 2A). In contrast, the *C3*^–/–^ mice were significantly protected from infection, with no significant weight loss evident at any time point. Surprisingly, viral titers in the lung were similar in *C3*^–/–^ and C57BL/6J controls (Fig. 2B), indicating that the lack of disease in *C3*^–/–^ mice is uncoupled from viral replication efficiency and that complement signaling is not necessary for SARS-CoV MA15 clearance from the lung. We further measured SARS-CoV MA15-induced disease by assessing respiratory function using whole-body plethysmography following infection of C57BL/6J and *C3*^–/–^ mice. Enhanced pause (Penh) is a calculated measure of airway resistance that we have associated with airway debris following SARS-CoV MA15 infection (24). The 50% exhalation force (EF50) measures the exhalation force midbreath, which increases as breathing becomes more difficult. Finally, the ratio of peak expiratory flow (RPEF) is the time to peak expiratory flow and has been associated with wheezing following infection. All three metrics have been shown to change significantly following SARS-CoV MA15 infection, with Penh and EF50 increasing following infection and RPEF decreasing. Combined, these measurements show that SARS-CoV MA15-infected animals have altered exhalation patterns in their breathing. *C3*^–/–^ mice exhibit a decreased change in Penh and EF50 levels following SARS-CoV MA15 infection relative to those of infected C57BL/6J control mice; however, RPEF values were similar between infection conditions (Fig. 2C to E). Together, these data indicate that despite the lack of weight loss in *C3*^–/–^ mice, the absence of the complement pathway did not alter host control of viral replication or completely abolish respiratory disease following SARS-CoV MA15 infection.

In order to determine which arm of the complement pathway contributes to SARS-CoV MA15 pathogenesis, we infected knockout mice lacking components upstream of C3. *C4*-deficient mice lack the ability to signal through both the classical and lectin pathways, while *fB*-deficient mice lack the ability to signal through the alternative pathway. Both mouse strains showed reduced weight loss relative to that of infected control mice (see Fig. S1 in the supplemental material) at 3 days postinfection; however, neither *C4*^–/–^ nor *fB*^–/–^ mice reproduced complete protection from weight loss observed in *C3*^–/–^ controls. Together, the results suggest that multiple arms of the complement pathway may be activated and contribute to SARS-CoV-mediated disease through C3 activation.

10.1128/mBio.01753-18.1 FIG S1 Complement pathway knockouts. *C4* and *fB* complement pathway-specific knockout mice were intranasally infected with 10^5^ PFU of MA15; both show an intermediate phenotype, with significantly less weight loss than that of C57BL/6J mice but more weight loss than that of *C3* knockout mice at day 3 postinfection. *, *P* < 0.05 between C57BL/6J and pathway-specific knockout mice; †, *P* < 0.05 between *C3*^–/–^ and pathway-specific knockout mice. Error bars indicate SEM, with 8 *C4*^–/–^ mice, 14 C57BL/6J mice, 7 *C3*^–/–^ mice, and 3 *fB*^–/–^ mice. Download FIG S1, TIF file, 0.45 MB. Copyright © 2018 Gralinski et al. 2018 Gralinski et al. This content is distributed under the terms of the Creative Commons Attribution 4.0 International license.

### Reduced lung pathology in *C3*^–/–^ mice.

**FIG 3:**
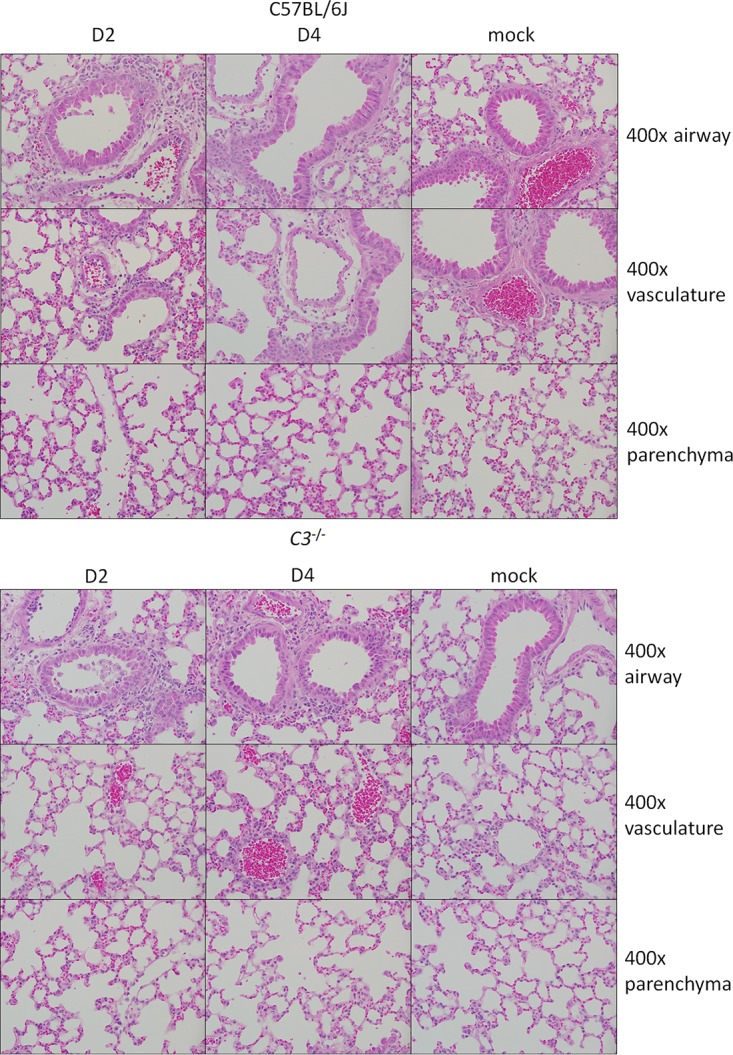
Histological analysis of C57BL/6J and *C3*^−/−^ lungs at 2 and 4 days postinfection. Representative images show 400× magnifications of the large airways (top row), vasculature (middle row), and parenchyma (bottom row) of the lung after SARS-CoV MA15 or mock infection of C57BL/6J or *C3*^−/−^ mice.

**FIG 4:**
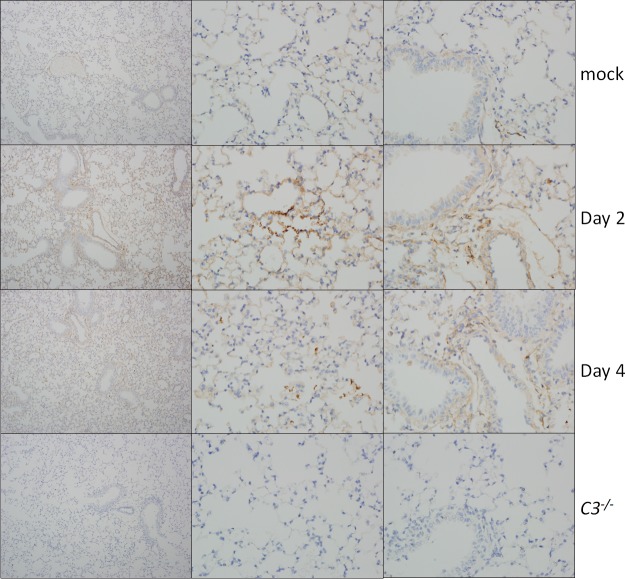
Complement deposition staining. Complement deposition on lung tissue of C57BL/6J mice (top three rows) was assessed by immunohistochemistry. Mice were examined at 2 and 4 dpi and mock infected or infected with SARS-CoV MA15. *C3*^–/–^ mice (bottom row) showed no positive staining.

Analysis of SARS-CoV MA15-infected lung sections showed that the absence of *C3* resulted in reduced, but still significant, lung pathology. At day 2 postinfection, only minor effects on lung disease were observed with airway denudation and debris, the main histopathological phenotypes at this early time point; levels of pathology were similar between wild-type and knockout mice (Fig. 3; Table S1). *C3*^–/–^ mice exhibited more airspace inflammation, including eosinophils, at 2 dpi than their wild-type controls, although this relationship was reversed later in infection. At 4 dpi, C57BL/6J mice displayed pronounced lung pathology, including inflammatory cells in the large airway and parenchyma, perivascular cuffing, thickening of the interstitial membrane, and low levels of intra-alveolar edema. In contrast, *C3*^–/–^ mice showed reduced scores in these areas, consistent with the improved respiratory function observed in Fig. 2. Notably, the lung pathology results were not as pronounced as the complete absence of weight loss, suggesting a possible distinction between lung disease and overall pathogenesis. We also considered whether the decrease in SARS-CoV MA15 pathogenesis in *C3*^–/–^ mice was due to reduced lung damage in the absence of complement pathway signaling. To investigate this possibility, we looked for signs of complement deposition on SARS-CoV MA15 lung tissue. At both 2 and 4 dpi, we observed scattered positive staining for complement in the lungs of SARS-CoV MA15-infected mice, suggesting that local tissue damage might contribute to SARS-CoV pathogenesis (Fig. 4). Interestingly, staining was consistently found in the parenchyma of the lung and not in the large airways, which are the other main site of SARS-CoV MA15 replication. No positive staining was observed in the lungs of *C3*^–/–^ mice.

10.1128/mBio.01753-18.3 TABLE S1 Histology scoring summary of C57BL/6J and *C3*^–/–^ mice at 2 and 4 days postinfection. Download Table S1, DOCX file, 0.01 MB. Copyright © 2018 Gralinski et al. 2018 Gralinski et al. This content is distributed under the terms of the Creative Commons Attribution 4.0 International license.

### Diminished infiltration of the lungs of a select immune population of infected *C3*^–/–^ mice.

**FIG 5:**
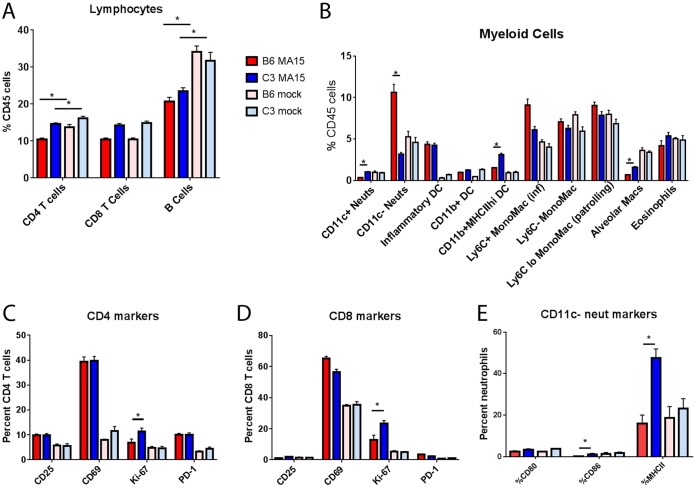
Inflammatory cells of C57BL/6J mice and *C3* knockout mice. Flow cytometric analysis of inflammatory cells present in the lungs of SARS-CoV MA15-infected or mock-infected C57BL/6J or *C3*^–/–^ mice at 4 days postinfection. (A) Lymphocytes; (B) myeloid-cell-derived cells (as defined by Misharin et al. [68]); (C) CD4 T cell activation markers; (D) CD8 T cell activation markers; (E) CD11c^–^ neutrophil activation markers. *, *P* < 0.05. Error bars indicate SEM. Eight mice were used for all infection groups, and 4 mice were used for all mock-infected groups. Neuts, neutrophils; DC, dendritic cells; MHCIIhi, a high fluorescence intensity for MHCII staining; MonoMac (inf), inflammatory monocyte-macrophages; Macs, macrophages.

In order to identify and quantitate inflammatory cells in the lung, we performed flow cytometry at 4 dpi. In parallel with humans exhibiting lung pathology, *C3*^–/–^ mice exhibited significant pulmonary infiltration following SARS-CoV MA15 infection, but this inflammation was reduced relative to that observed in wild-type mice. SARS-CoV MA15-infected C57BL/6J and *C3*^–/–^ mice had similar total cell counts as well as similar percentages of CD45-positive cells in their lungs (data not shown). Consistently with what was observed in human SARS-CoV patients (25), lymphopenia was observed in the lungs of both SARS-CoV MA15-infected C57BL/6J and *C3*^–/–^ mice with reduced percentages of B cells (Fig. 5A) and CD4 T cells relative to those in mock-infected mice following infection. Despite similar overall lymphocyte levels, small but significant differences were observed in levels of T cell activation between infected C57BL/6J and *C3*^–/–^ mice; both CD4 and CD8 T cells in *C3*^–/–^ mice expressed more Ki-67 (Fig. 5C and D), an intracellular marker of proliferation, than those in C57BL/6J controls. Analysis of myeloid cells in the lung showed that infected C56BL/6J mice had significantly higher levels of neutrophils, particularly nonactivated neutrophils, in the lung than infected *C3*^–/–^ mice (Fig. 5B and E). Furthermore, inflammatory monocytes, which have previously been associated with increased SARS-CoV MA15 pathogenesis (26), were significantly increased in the lungs of wild-type mice but not *C3*^–/–^ mice (Fig. 5B). Finally, we observed significantly more dendritic cells and alveolar macrophages in the lungs of SARS-CoV MA15-infected *C3*^–/–^ mice than in the lungs of infected C57BL/6J mice. Together, although *C3*^–/–^ mice produced a robust immune cell infiltration following SARS-CoV infection, they had significant reductions in both inflammatory monocytes and neutrophils relative to controls; both cell types that are associated with SARS-CoV pathogenesis (27). Conversely, the presence of activated T cells is associated with recovery following infection (28).

In addition to examining inflammatory cells, we evaluated the vascular integrity of the lung following SARS-CoV MA15 infection in the presence and absence of *C3*. We observed no differences in the numbers of platelets present in the bronchoalveolar lavage (BAL) fluid between C57BL/6J and *C3*^–/–^ mice at either 2 or 4 days postinfection, indicating that the absence of *C3* does not appear to significantly alter vascular permeability following infection with SARS-CoV (Fig. S2B).

10.1128/mBio.01753-18.2 FIG S2 Bronchoalveolar lavage. Platelet counts in BAL fluid were assessed using a VetscanHM5 at both day 2 and day 4 post infection. (Four mice were used for all infected groups, and 2 mice were used for all mock-infected groups.) Download FIG S2, TIF file, 0.96 MB. Copyright © 2018 Gralinski et al. 2018 Gralinski et al. This content is distributed under the terms of the Creative Commons Attribution 4.0 International license.

### Cytokine and chemokine levels are significantly decreased in the lungs of *C3*^–/–^ mice.

**FIG 6:**
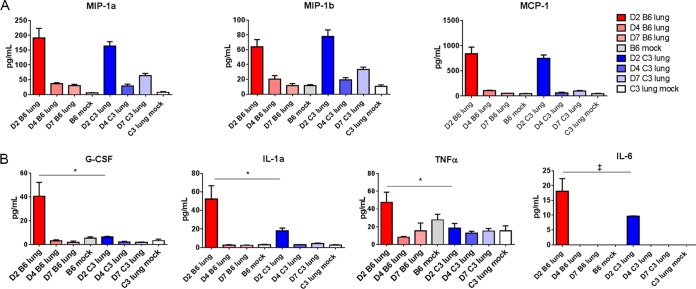
Cytokine and chemokine abundance levels. Protein abundance in the lung was measured by Bioplex multiplex magnetic bead assay at days 2, 4, and 7 postinfection or in mock-infected mice. MIP-1a, MIP-1b, and monocyte chemoattractant protein (MCP) had similar concentrations in the lungs of C57BL/6J and *C3*^–/–^ mice, with peak abundance at 2 days postinfection (A), while G-CSF, IL-6, TNF, and IL-1a expression was highest in C57BL/6J mice at 2 dpi (B). *, *P* < 0.05; ‡, *P* < 0.1. Error bars indicate SEM, and 3 to 4 mice were tested for each condition.

To further investigate the inflammatory response to SARS-CoV MA15 infection, we measured cytokine and chemokine protein levels in the lung in the presence and absence of complement signaling. Multiple protein expression patterns were observed in response to infection. MIP1a, MIP1b, and MCP1 are all highly expressed in the lung following SARS-CoV MA15 infection of both C57BL/6J and *C3*^–/–^ mice (Fig. 6A), indicating that some inflammatory signaling remains intact in the absence of *C3*. Granulocyte colony-stimulating factor (G-CSF), interleukin 6 (IL-6), tumor necrosis factor alpha (TNF-α), and IL-1a comprised a group of cytokines and chemokines that were more highly produced in the lungs of C57BL/6J mice than in *C3*^–/–^ mice (Fig. 6B), all peaking at 2 days postinfection. Notably, these cytokines all have a role in the production, recruitment, or differentiation of neutrophils, consistent with the flow cytometry results in Fig. 5B. With the exception of RANTES, all inflammatory cytokines and chemokines were measured at the highest levels at 2 dpi, indicating that the host immune response is triggered quickly following infection with SARS-CoV MA15. Together, these results indicate that the absence of complement has an impact on the magnitude of some cytokines and chemokines in the lung; however, robust production can occur in either the presence or the absence of *C3*.

### SARS-CoV MA15 induces systemic complement activation.

**FIG 7:**
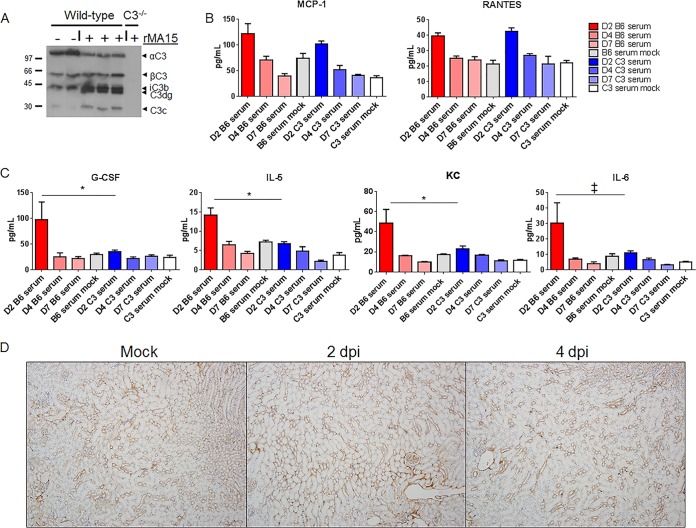
Systemic response to SARS-CoV MA15 infection. (A) C3 protein cleavage products are observed by Western blotting in the serum following infection. Molecular weights are noted at the left (in thousands). rMA15, recombinant MA15. (B) MCP-1 and RANTES levels are similarly elevated following infection in C57BL/6J and *C3*^–/–^ mice. (C) G-CSF, KC, and IL-5 all have significantly higher expression in the sera of C57BL/6J mice than in those of C3^–/–^ mice. IL-6 expression was suggestive of differences. (D) Complement deposition staining in the kidneys of C57BL/6J mice. *, *P* < 0.05; ‡, *P* < 0.06. Error bars indicate SEM, and 3 to 4 mice were used for each condition.

The absence of complement signaling resulted in reduced SARS-CoV MA15 pathogenesis, as measured by weight loss and a partial reduction of respiratory dysfunction, pathology, immune infiltration, and cytokine responses in the lung. We hypothesized that systemic disease coupled with no change in viral titer might also drive important elements of complement-mediated disease. Therefore, we examined sera from wild-type and *C3*^–/–^ mice for signs of systemic disease following infection. Western blot analysis showed increased levels of C3a-derived fragments in the serum, indicating systemic complement activation in SARS-CoV MA15-infected mice at 2 dpi (Fig. 7A). Given this result, we next examined cytokine and chemokine protein levels for markers of inflammation in the sera of SARS-CoV MA15-infected mice. Both MCP-1 and RANTES levels were elevated in the serum following infection, regardless of mouse genetic background (Fig. 7B). However, numerous cytokines and chemokines, such as IL-5, G-CSF, and KC (keratinocyte chemoattractant or CXCL1) were present in significantly higher abundance in the lungs of C57BL/6J mice than in those of *C3* knockout mice (Fig. 7C). We further examined the possibility that SARS-CoV MA15 infection leads to complement deposition outside the lung and found no signs of increased complement staining in the kidney (Fig. 7D). Although no complement deposition was seen, the presence of both activated complement and inflammatory cytokines in the sera likely contributes to a systemic inflammatory response that drives SARS-CoV MA15-mediated weight loss following infection.

## DISCUSSION

The complement system is a critical part of the host immune response to bacterial and viral infection. Originally identified in the 1900s as a heat-sensitive, nonspecific complement to the more specific adaptive immune pathways (29), the complement system is one way that the innate immune system detects and responds to foreign antigens. Because of its potential to damage host tissues, the complement system is also tightly regulated through a number of inhibiting proteins that are constitutively present in the serum (30). It has previously been shown that complement pathway signaling is critical for the protective host immune response to various bacterial infections (31) as well as some influenza virus and flavivirus infections (22, 32, 33). Furthermore, viruses, including herpesviruses, poxviruses, astroviruses, flaviviruses, and retroviruses, encode genes to help them evade detection by the complement system (17), strong evidence that complement is important in the host antiviral response. The host factors that drive protective (22) or pathogenic (34) complement-associated responses in viral infection are not well understood. Of particular concern, the anaphylatoxins C3a, C4a, and C5a are produced during activation of the complement signaling cascade; they have potent proinflammatory properties and can trigger inflammatory cell recruitment and neutrophil activation (35). C3a and C5a blockade has been proposed as a treatment for acute lung injury (36), and anti-C5a antibody has been shown to protect mice from infection with influenza virus (34) and, more recently, MERS-CoV (16). Complement recognition is important for the control of paramyxoviruses (37), dengue virus (38), and human T lymphotrophic virus type 1 (HTLV-1) (39), and many more viruses have developed means of evading detection by the complement system (17). In contrast, the data presented here, in conjunction with recent findings for Ross River virus (40, 41), influenza virus (34), and well-established autoimmune disease (42), demonstrate that complement system activation can also lead to exacerbated disease.

Previous reports clearly established the ability of mannose-binding lectin (MBL) to bind to the SARS-CoV spike protein (11), dependent on an N-linked glycosylation site; however, the role of complement signaling in SARS-CoV pathogenesis was unclear (9, 10, 13, 43). In this study, we demonstrate that the complement system is activated following SARS-CoV MA15 infection (Fig. 1B). However, we did not observe any change in viral titer in *C3*^–/–^ mice (Fig. 2B), indicating an important difference between *in vivo* and *in vitro* studies and the use of viral pseudoparticles. The absence of complement signaling resulted in protection from SARS-CoV MA15-induced weight loss, as shown through the use of both *C3*-deficient mice (Fig. 2A) and activation pathway-specific knockout mice (Fig. S1). Respiratory function in *C3* knockout mice was improved relative to that of control mice, although significant changes in Penh and RPEF were still observed, indicating that the elimination of complement signaling did not completely remove the effects of SARS-CoV MA15 infection. While analysis of the cellular inflammatory response to SARS-CoV MA15 infection revealed modest changes in histopathology and overall inflammatory cell recruitment to the lungs, significant differences were observed in pathogenic inflammatory monocyte and neutrophil populations, indicating that complement signaling contributes to the broader immune response to infection. Immunohistochemical staining revealed that SARS-CoV MA15 infection induced complement deposition in the lung (Fig. 4), similar to that associated with pathogenesis in Ross River virus-infected mice (41) and some influenza virus infections (34), and it is likely that complement deposition contributes to pulmonary disease and inflammatory cell recruitment.

The cytokines and chemokines IL-5, IL-6, KC (CXCL1), and G-CSF have higher abundances in the lungs of SARS-CoV MA15-infected wild-type mice than in *C3*^–/–^ mice and are all known to promote neutrophil recruitment. Indeed, significantly more neutrophils were observed in the lungs of SARS-CoV MA15-infected C56BL/6J mice than in the *C3*^–/–^ mice (Fig. 5B). Interestingly, while there were fewer neutrophils present, the neutrophils found in the lungs of *C3*^–/–^ mice infected with SARS-CoV MA15 had significantly more staining of major histocompatibility complex class II (MHC II) and the costimulatory molecules CD80 and CD86 (Fig. 5E), indicating a state of activation (44). Unpublished data from our laboratory have consistently demonstrated higher neutrophil counts in the lungs of mice with severe disease than in those of mice with only mild pathogenesis. Additionally, neutrophilia in human SARS-CoV patients was associated with a poor outcome of infection (45), and studies of native rat coronavirus (46) found both a protective and a pathogenic role for neutrophils following infection. Combined, these data demonstrate that the absence of complement provides significant improvements in pulmonary disease following SARS-CoV MA15 infection and suggest that a nonpulmonary cause might also contribute to the lack of weight loss in *C3*^–/–^ mice.

Importantly, our data demonstrate that SARS-CoV MA15 infection activates the complement system systematically as well as in the lung (Fig. 7A and 1B). Wild-type C57BL/6J mice exhibited an increased abundance of serum cytokines and chemokines in response to SARS-CoV MA15 infection (Fig. 7C) in comparison to *C3*^–/–^ mice. In particular, the pyrogenic cytokine IL-6 (47) is present at higher abundance in the lungs and sera of C57BL/6J mice than in those of *C3*^–/–^ mice. IL-1α and TNF-α are also more abundant in the lungs of C57BL/6J mice, suggesting that wild-type but not *C3*^–/–^ mice develop a fever response to infection that contributes to weight loss and respiratory dysfunction phenotypes. While the precise mechanism of complement activation following SARS-CoV MA15 infection is still unclear, it is likely through recognition of the viral spike glycoprotein and partially mediated by MBL (see Fig. S1 in the supplemental material).

The anaphylatoxins produced by the activated complement pathway, C4a, C3a, and C5a, have important immunostimulatory roles in vascular permeability and inflammatory cell recruitment (35, 48). C3a and C5a in particular are noted for their roles in causing mast cell degranulation, initiating a cytokine storm, promoting vascular permeability, and contributing to acute lung injury (36, 49, 50). Furthermore, a C5a antibody blockade was recently shown to protect in a model of highly susceptible MERS-CoV mice (16). Although there are no reports of mast cell activation following SARS-CoV or MERS-CoV infection, activation has been observed both *in vitro* and *in vivo* following infection with influenza virus (51) and may occur following other severe respiratory infections, including coronaviruses. Mast cells release cytokines, including IL-6, IL-1, and TNF-α, consistent with the inflammatory profile observed following SARS-CoV MA15 infection. While this work cannot definitively conclude that the complement anaphylatoxins and mast cell activation contribute to SARS-CoV MA15 pathogenesis, the data are consistent with this possibility, and the concept warrants further investigation.

Complement pathway activation is a hallmark of bacterial infection, and genetic deficiencies in the complement pathway result in enhanced susceptibility to Streptococcus pneumoniae, Neisseria meningitidis, and *Haemophilus influenzae* infections (52), as well as to sepsis. Interestingly, it has also been shown that SARS-CoV MA15 infection stimulates TLR4 (53, 54), which is classically known as the lipopolysaccharide (LPS) receptor (55, 56) and important for recognition of many bacterial infections. Combined, these data suggest that host recognition of SARS-CoV MA15 infection may activate similar pathways recognizing a bacterial infection, leading to immune signaling cascades that cause systemic disease and enhance viral pathogenesis. While systemic activation of the complement pathway may be useful during a bacterial infection, it is less so during a localized acute viral infection, such as SARS-CoV. Furthermore, complement activation, in conjunction with the presence of neutrophils, is known to cause increased vascular permeability, a condition that is also observed following SARS-CoV infection (57–59) and was associated with poor outcome. Baseline complement activation also increases with age (60, 61), consistent with increased SARS-CoV morbidity and mortality in aged populations. Finally, it has previously been reported that serum C5a levels are predictive of ARDS development (62) and that, in the absence of complement, animals are protected from bacterially induced “shock lung” (63), data consistent with the pathogenic role that we have found for complement following SARS-CoV MA15 infection.

In this work, we demonstrate that SARS-CoV MA15 infection activates the complement pathway and that complement signaling contributes to disease following infection. This disease is likely mediated by complement protein deposition in the lung as well as systemic complement activation and inflammation. Notably, the absence of *C3* has no impact on viral titer, unlike what has been observed following influenza virus infection. Despite these differences, it is notable that MERS-CoV and H5N1 influenza virus-induced acute lung injury and pulmonary inflammation are reduced in mice that are treated with either a C3a receptor (C3aR) antagonist or antibodies to C5a (16, 34). A similar treatment might be effective in mitigating SARS-CoV MA15-induced disease, as SARS, MERS, and influenza have common disease manifestations, including development of acute lung injury. Given the large array of zoonotic strains poised for cross-species transmission, broad-based inhibitors of emerging coronavirus infections are a high priority (64). Pinpointing the precise arms of the complement pathway that contribute to SARS-CoV will help further identify therapeutic targets while minimizing unnecessary inhibition of the immune response. This work suggests that investigation of anticomplement drugs for treatment of coronavirus infections is warranted and would pair well with direct antiviral therapeutics.

## MATERIALS AND METHODS

### Viruses and cells.

Stocks of recombinant mouse-adapted SARS-CoV (MA15) (65) were propagated and their titers were determined in Vero E6 cells and stored as single-use aliquots at −80°C as previously described (66). Tissue titers of MA15 were determined by plaque assay on Vero E6 cells as previously described (66, 67), with a limit of detection of 100 PFU. All experiments using live virus were performed in a class II biological safety cabinet in a certified biosafety level 3 laboratory with negative air pressure and redundant exhaust fans; personnel wore personal protective equipment, including Tyvek suits, hoods, and powered air-purifying respirators.

### Mouse experiments.

C57BL/6J (stock number 000664) and *C3*^–/–^ (stock number 003641) mice were purchased from Jackson Laboratories. *fB^−/−^* mice were generously provided by Charles Jennette (UNC), and *C4*^–/–^ mice were provided by Mark Heise (UNC). All animal husbandry and experiments were performed in accordance with all University of North Carolina at Chapel Hill Institutional Animal Care and Use Committee guidelines. Age-matched (10- to 11-week-old) female mice were anesthetized with a mixture of ketamine-xylazine and intranasally inoculated with 50 μl of phosphate-buffered saline (PBS) or 10^5^ PFU of SARS-CoV MA15 diluted in PBS. Mice were monitored for disease signs and weighed at 24-h intervals.

### Microarray and proteomics analysis.

Microarray and proteomics analyses were performed on a time course and according to a dose-response study published by Gralinski et al. by following the same methods (23). The proteomics data (experiment SM001) are publically available through the PNNL (http://omics.pnl.gov) Web portal. Briefly the mean intensity (abundance) for each protein was then graphed as an average (5 mice for each infection, 3 mice for mock infection) for each group at each time point. Missing or absent values were not scored; however, if no value was observed in any of the samples at a time point, the sample was registered with a single 0, representing “not detected.”

### Histological analysis.

At the times indicated in the figures, mice were euthanized using an overdose of isoflurane, and lung tissue was fixed in 10% formalin. Tissues were embedded in paraffin, and 5-μm sections were prepared. To determine the extent of inflammation and tissue pathology, tissues were stained with hematoxylin and eosin and scored in a blind manner from 0 (no sign of phenotype) to 3 (widespread and severe phenotype).

### Platelet counts.

For bronchoalveolar lavage (BAL), immediately following euthanasia, 1 ml of PBS was injected into the lung through the trachea by using a 22-gauge Exel Safelet catheter tip (Fisher). This fluid was then drawn back out and used for subsequent analysis. Two hundred fifty microliters of BAL fluid was used for absolute counting of gross cell types using an Abaxis VetScan HM5 analyzer.

### Complement deposition staining.

Lung sections from SARS-CoV MA15-, Ross River virus-, or mock-infected mice were stained for the presence of C3 by the Animal Histopathology and Laboratory Medicine Core at the University of North Carolina. SARS-CoV MA15 lung samples were tested from 20-week-old mice at 1, 2, 4, and 7 days after infection with 10^5^ PFU of virus. Staining was performed using a goat anti-mouse C3 primary antibody (MP Biomedicals).

### Immunoblot analysis.

Mice were perfused with PBS, and then lung tissue was dissected and homogenized in lysis buffer (50 mM Tris [pH 8.0], 150 mM NaCl, 1% Nonidet P-40, 0.5% deoxycholate, and 0.1% sodium dodecyl sulfate [SDS] supplemented with Complete protease inhibitor cocktail [Roche]). Total protein concentrations were determined by using the Coomassie Plus assay kit (Pierce). Dilutions of serum or 20- to 30-μg aliquots of protein were diluted in an equal volume of 2× SDS sample buffer, and SDS-polyacrylamide gel electrophoresis was performed. Proteins were transferred onto polyvinylidene fluoride membranes (Bio-Rad). Membranes were blocked in 1× PBS–5% milk–0.1% Tween 20 and incubated with goat anti-mouse C3 antibody (1:1,000; Cappel) overnight at 4°C. Membranes were washed in PBS–0.1% Tween 20 and incubated with rabbit anti-goat-horseradish peroxidase (1:10,000; Sigma) for 1 h at room temperature. After a washing step, proteins were visualized by enhanced chemiluminescence (Amersham) according to the manufacturer’s instructions.

### Flow cytometry.

Following euthanasia at 4 days postinfection, mice were perfused with 10 ml of PBS via cardiac puncture. Lungs were dissected, minced, and incubated for 90 min with vigorous shaking at 37°C in digestion buffer (RPMI 1640, 10% fetal bovine serum [FBS], 15 mM HEPES, 2.5 mg/ml collagenase A [Worthington], 1.7 mg/ml DNase I [Sigma]). Enzymatically digested tissues were passed through a 70-μm cell strainer and pelleted, and red blood cells were lysed using ammonium-chloride-potassium (ACK) lysis buffer. Total numbers of viable cells were determined by trypan blue exclusion. Isolated cells were stained in fluorescence-activated cell sorting (FACS) staining buffer (1× Hanks balanced salt solution [HBSS], 1% FBS) with the following antibodies: CD45-leukocyte common antigen (LCA)-allophycocyanin (APC) eFlour780 (clone 30-F11; eBioscience), CD3-peridinin chlorophyll protein (PerCP) Cy5.5 (clone 145-2C11; eBioscience), CD4-BUV737 (clone RMA4-5; BD), CD8-phycoerythrin (PE) (clone 53-6.7; BD), CD19-BV650 (clone 6D5; BioLegend), CD25-BV510 (clone PC61; BD), CD44-BV786 (clone IM7; BD), CD62L-BUV395 (clone MEL-14; BD), Ki-67–fluorescein isothiocyanate (FITC) (clone SolA15; eBioscience), CD69-PECF594 (clone H1.2F3; BD), NK1.1-PE Cy7 (clone PK136; eBioscience), PD-1-BV605 (clone J43; BD), CD45-LCA–APC-R700 (clone 30-F11; BD), CD11b-BV785 (clone M1/70; BioLegend), CD11c-PECF594 (clone HL3; BD), MHC II-APC (clone M5/114.15.2; eBioscience), Ly6G-APC-Fire780 (clone 1A8; BioLegend), Ly6C-BV605 (clone AL-21; BD), SiglecF-BV650 (clone E50-2440; BD), CD80-BUV737 (clone 16-10A1; BD), CD86-BV421 (clone GL1; BD), and CD103-PerCP-eFluor710 (clone 2E7; eBioscience). After being stained, cells were fixed in 2% paraformaldehyde overnight and then stored in PBS until acquisition within 24 h. A minimum of 100,000 events were collected using an LSRII cytometer (Becton, Dickinson), and analysis was completed using FlowJo software version 10 (TreeStar). All samples were first evaluated through subsequent gates for (i) mononuclear cells, (ii) doublet exclusion, (iii) dead-cell exclusion based on uptake of a fixable live/dead cell discriminator (Invitrogen), and CD45-LCA expression before downstream analyses. Reported cell frequencies were normalized to the percentage of total CD45-LCA^+^ events, where appropriate.

### Whole-body plethysmography.

Respiratory function was measured using whole-body plethysmography as described by Menachery et al. (24). Briefly, mice were loaded into individual chambers and allowed to acclimate for 30 min before a 5-min measurement window. Measurements were recorded every 2 s for a total of 150 measurements per time point per mouse.

### Cytokine and chemokine protein analysis.

The small center lung lobe of each mouse was homogenized in 1 ml of PBS and briefly centrifuged to remove debris. Fifty microliters of homogenate was used to measure cytokine and chemokine protein abundance using a Bio-Plex Pro mouse cytokine 23-plex assay (Bio-Rad) according to the manufacturer’s instructions.

### Statistical analyses.

Percent starting body weights, viral titers, and inflammatory cell numbers were evaluated for statistically significant differences by the Mann-Whitney test or Student’s *t* test using GraphPad Prism software.

### Accession number(s).

The microarray data were previously deposited in the GEO database under accession number GSE33266 (23).

## References

[1] Chinese SARS Molecular Epidemiology Consortium. 2004 Molecular evolution of the SARS coronavirus during the course of the SARS epidemic in China. Science 303:1666–1669. doi:10.1126/science.1092002. 14752165

[2] Rota PA, Oberste MS, Monroe SS, Nix WA, Campagnoli R, Icenogle JP, Peñaranda S, Bankamp B, Maher K, Chen MH, Tong S, Tamin A, Lowe L, Frace M, DeRisi JL, Chen Q, Wang D, Erdman DD, Peret TC, Burns C, Ksiazek TG, Rollin PE, Sanchez A, Liffick S, Holloway B, Limor J, McCaustland K, Olsen-Rasmussen M, Fouchier R, Günther S, Osterhaus AD, Drosten C, Pallansch MA, Anderson LJ, Bellini WJ 2003 Characterization of a novel coronavirus associated with severe acute respiratory syndrome. Science 300:1394–1999. doi:10.1126/science.1085952. 12730500

[3] Christian MD, Poutanen SM, Loutfy MR, Muller MP, Low DE 2004 Severe acute respiratory syndrome. Clin Infect Dis 38:1420–1427. doi:10.1086/420743. 15156481 PMC7107873

[4] Zaki AM, van Boheemen S, Bestebroer TM, Osterhaus AD, Fouchier RA 2012 Isolation of a novel coronavirus from a man with pneumonia in Saudi Arabia. N Engl J Med 367:1814–1820. doi:10.1056/NEJMoa1211721. 23075143

[5] Hui DS, Memish ZA, Zumla A 2014 Severe acute respiratory syndrome vs. the Middle East respiratory syndrome. Curr Opin Pulm Med 20:233–241. doi:10.1097/MCP.0000000000000046. 24626235

[6] Menachery VD, Yount BL Jr, Debbink K, Agnihothram S, Gralinski LE, Plante JA, Graham RL, Scobey T, Ge XY, Donaldson EF, Randell SH, Lanzavecchia A, Marasco WA, Shi ZL, Baric RS 2015 A SARS-like cluster of circulating bat coronaviruses shows potential for human emergence. Nat Med 21:1508–1513. doi:10.1038/nm.3985. 26552008 PMC4797993

[7] Ge XY, Li JL, Yang XL, Chmura AA, Zhu G, Epstein JH, Mazet JK, Hu B, Zhang W, Peng C, Zhang YJ, Luo CM, Tan B, Wang N, Zhu Y, Crameri G, Zhang SY, Wang LF, Daszak P, Shi ZL 2013 Isolation and characterization of a bat SARS-like coronavirus that uses the ACE2 receptor. Nature 503:535–538. doi:10.1038/nature12711. 24172901 PMC5389864

[8] Cheng VC, Lau SK, Woo PC, Yuen KY 2007 Severe acute respiratory syndrome coronavirus as an agent of emerging and reemerging infection. Clin Microbiol Rev 20:660–694. doi:10.1128/CMR.00023-07. 17934078 PMC2176051

[9] Ip WK, Chan KH, Law HK, Tso GH, Kong EK, Wong WH, To YF, Yung RW, Chow EY, Au KL, Chan EY, Lim W, Jensenius JC, Turner MW, Peiris JS, Lau YL 2005 Mannose-binding lectin in severe acute respiratory syndrome coronavirus infection. J Infect Dis 191:1697–1704. doi:10.1086/429631. 15838797 PMC7199483

[10] Yuan FF, Tanner J, Chan PK, Biffin S, Dyer WB, Geczy AF, Tang JW, Hui DS, Sung JJ, Sullivan JS 2005 Influence of FcγRIIA and MBL polymorphisms on severe acute respiratory syndrome. Tissue Antigens 66:291–296. doi:10.1111/j.1399-0039.2005.00476.x. 16185324 PMC7190181

[11] Zhou Y, Lu K, Pfefferle S, Bertram S, Glowacka I, Drosten C, Pohlmann S, Simmons G 2010 A single asparagine-linked glycosylation site of the severe acute respiratory syndrome coronavirus spike glycoprotein facilitates inhibition by mannose-binding lectin through multiple mechanisms. J Virol 84:8753–8764. doi:10.1128/JVI.00554-10. 20573835 PMC2919028

[12] Leth-Larsen R, Zhong F, Chow VT, Holmskov U, Lu J 2007 The SARS coronavirus spike glycoprotein is selectively recognized by lung surfactant protein D and activates macrophages. Immunobiology 212:201–211. doi:10.1016/j.imbio.2006.12.001. 17412287 PMC7114820

[13] Wang Y, Yan J, Shi Y, Li P, Liu C, Ma Q, Yang R, Wang X, Zhu L, Yang X, Cao C 2009 Lack of association between polymorphisms of MASP2 and susceptibility to SARS coronavirus infection. BMC Infect Dis 9:51. doi:10.1186/1471-2334-9-51. 19405982 PMC2683852

[14] Mathern DR, Heeger PS 2015 Molecules great and small: the complement system. Clin J Am Soc Nephrol 10:1636–1650. doi:10.2215/CJN.06230614. 25568220 PMC4559511

[15] Ricklin D, Hajishengallis G, Yang K, Lambris JD 2010 Complement: a key system for immune surveillance and homeostasis. Nat Immunol 11:785–797. doi:10.1038/ni.1923. 20720586 PMC2924908

[16] Jiang Y, Zhao G, Song N, Li P, Chen Y, Guo Y, Li J, Du L, Jiang S, Guo R, Sun S, Zhou Y 2018 Blockade of the C5a-C5aR axis alleviates lung damage in hDPP4-transgenic mice infected with MERS-CoV. Emerg Microbes Infect 7:77. doi:10.1038/s41426-018-0063-8. 29691378 PMC5915580

[17] Stoermer KA, Morrison TE 2011 Complement and viral pathogenesis. Virology 411:362–373. doi:10.1016/j.virol.2010.12.045. 21292294 PMC3073741

[18] Sarma VJ, Huber-Lang M, Ward PA 2006 Complement in lung disease. Autoimmunity 39:387–394. doi:10.1080/08916930600739456. 16923538

[19] Chang WC, White MR, Moyo P, McClear S, Thiel S, Hartshorn KL, Takahashi K 2010 Lack of the pattern recognition molecule mannose-binding lectin increases susceptibility to influenza A virus infection. BMC Immunol 11:64. doi:10.1186/1471-2172-11-64. 21182784 PMC3022599

[20] Thielens NM, Tacnet-Delorme P, Arlaud GJ 2002 Interaction of C1q and mannan-binding lectin with viruses. Immunobiology 205:563–574. doi:10.1078/0171-2985-00155. 12396016

[21] Bera MM, Lu B, Martin TR, Cui S, Rhein LM, Gerard C, Gerard NP 2011 Th17 cytokines are critical for respiratory syncytial virus-associated airway hyperreponsiveness through regulation by complement C3a and tachykinins. J Immunol 187:4245–4255. doi:10.4049/jimmunol.1101789. 21918196 PMC3186836

[22] O'Brien KB, Morrison TE, Dundore DY, Heise MT, Schultz-Cherry S 2011 A protective role for complement C3 protein during pandemic 2009 H1N1 and H5N1 influenza A virus infection. PLoS One 6:e17377. doi:10.1371/journal.pone.0017377. 21408070 PMC3052313

[23] Gralinski LE, Bankhead A III, Jeng S, Menachery VD, Proll S, Belisle SE, Matzke M, Webb-Robertson BJ, Luna ML, Shukla AK, Ferris MT, Bolles M, Chang J, Aicher L, Waters KM, Smith RD, Metz TO, Law GL, Katze MG, McWeeney S, Baric RS 2013 Mechanisms of severe acute respiratory syndrome coronavirus-induced acute lung injury. mBio 4:e00271-13. doi:10.1128/mBio.00271-13. 23919993 PMC3747576

[24] Menachery VD, Gralinski LE, Baric RS, Ferris MT 2015 New metrics for evaluating viral respiratory pathogenesis. PLoS One 10:e0131451. doi:10.1371/journal.pone.0131451. 26115403 PMC4482571

[25] Lee N, Hui D, Wu A, Chan P, Cameron P, Joynt GM, Ahuja A, Yung MY, Leung CB, To KF, Lui SF, Szeto CC, Chung S, Sung JJ 2003 A major outbreak of severe acute respiratory syndrome in Hong Kong. N Engl J Med 348:1986–1994. doi:10.1056/NEJMoa030685. 12682352

[26] Channappanavar R, Fehr AR, Vijay R, Mack M, Zhao J, Meyerholz DK, Perlman S 2016 Dysregulated type I interferon and inflammatory monocyte-macrophage responses cause lethal pneumonia in SARS-CoV-infected mice. Cell Host Microbe 19:181–193. doi:10.1016/j.chom.2016.01.007. 26867177 PMC4752723

[27] van den Brand JM, Haagmans BL, van Riel D, Osterhaus AD, Kuiken T 2014 The pathology and pathogenesis of experimental severe acute respiratory syndrome and influenza in animal models. J Comp Pathol 151:83–112. doi:10.1016/j.jcpa.2014.01.004. 24581932 PMC7094469

[28] Zhao J, Zhao J, Perlman S 2010 T cell responses are required for protection from clinical disease and for virus clearance in SARS-CoV-infected mice. J Virol 84:9318–9932. doi:10.1128/JVI.01049-10. 20610717 PMC2937604

[29] Nesargikar PN, Spiller B, Chavez R 2012 The complement system: history, pathways, cascade and inhibitors. Eur J Microbiol Immunol 2:103–111. doi:10.1556/EuJMI.2.2012.2.2. PMC3956958 24672678

[30] Merle NS, Church SE, Fremeaux-Bacchi V, Roumenina LT 2015 Complement system part I—molecular mechanisms of activation and regulation. Front Immunol 6:262. doi:10.3389/fimmu.2015.00262. 26082779 PMC4451739

[31] Berends ET, Kuipers A, Ravesloot MM, Urbanus RT, Rooijakkers SH 2014 Bacteria under stress by complement and coagulation. FEMS Microbiol Rev 38:1146–1171. doi:10.1111/1574-6976.12080. 25065463

[32] Fuchs A, Lin TY, Beasley DW, Stover CM, Schwaeble WJ, Pierson TC, Diamond MS 2010 Direct complement restriction of flavivirus infection requires glycan recognition by mannose-binding lectin. Cell Host Microbe 8:186–195. doi:10.1016/j.chom.2010.07.007. 20709295 PMC2929649

[33] Mehlhop E, Diamond MS 2006 Protective immune responses against West Nile virus are primed by distinct complement activation pathways. J Exp Med 203:1371–1381. doi:10.1084/jem.20052388. 16651386 PMC2121216

[34] Sun S, Zhao G, Liu C, Wu X, Guo Y, Yu H, Song H, Du L, Jiang S, Guo R, Tomlinson S, Zhou Y 2013 Inhibition of complement activation alleviates acute lung injury induced by highly pathogenic avian influenza H5N1 virus infection. Am J Respir Cell Mol Biol 49:221–230. doi:10.1165/rcmb.2012-0428OC. 23526211

[35] Bosmann M, Ward PA 2012 Role of C3, C5 and anaphylatoxin receptors in acute lung injury and in sepsis. Adv Exp Med Biol 946:147–159. doi:10.1007/978-1-4614-0106-3_9. 21948367 PMC3372066

[36] Wang R, Xiao H, Guo R, Li Y, Shen B 2015 The role of C5a in acute lung injury induced by highly pathogenic viral infections. Emerg Microbes Infect 4:e28. doi:10.1038/emi.2015.28. 26060601 PMC4451266

[37] Johnson JB, Capraro GA, Parks GD 2008 Differential mechanisms of complement-mediated neutralization of the closely related paramyxoviruses simian virus 5 and mumps virus. Virology 376:112–123. doi:10.1016/j.virol.2008.03.022. 18440578 PMC2398685

[38] Avirutnan P, Hauhart RE, Marovich MA, Garred P, Atkinson JP, Diamond MS 2011 Complement-mediated neutralization of dengue virus requires mannose-binding lectin. mBio 2:e00276-11. doi:10.1128/mBio.00276-11. 22167226 PMC3236064

[39] Ikeda F, Haraguchi Y, Jinno A, Iino Y, Morishita Y, Shiraki H, Hoshino H 1998 Human complement component C1q inhibits the infectivity of cell-free HTLV-I. J Immunol 161:5712–5719. 9820553

[40] Morrison TE, Fraser RJ, Smith PN, Mahalingam S, Heise MT 2007 Complement contributes to inflammatory tissue destruction in a mouse model of Ross River virus-induced disease. J Virol 81:5132–5143. doi:10.1128/JVI.02799-06. 17314163 PMC1900244

[41] Gunn BM, Morrison TE, Whitmore AC, Blevins LK, Hueston L, Fraser RJ, Herrero LJ, Ramirez R, Smith PN, Mahalingam S, Heise MT 2012 Mannose binding lectin is required for alphavirus-induced arthritis/myositis. PLoS Pathog 8:e1002586. doi:10.1371/journal.ppat.1002586. 22457620 PMC3310795

[42] Chen M, Daha MR, Kallenberg CG 2010 The complement system in systemic autoimmune disease. J Autoimmun 34:J276–J286. doi:10.1016/j.jaut.2009.11.014. 20005073

[43] Zhang H, Zhou G, Zhi L, Yang H, Zhai Y, Dong X, Zhang X, Gao X, Zhu Y, He F 2005 Association between mannose-binding lectin gene polymorphisms and susceptibility to severe acute respiratory coronavirus infection. J Infect Dis 192:1355–1361. doi:10.1086/491479. 16170752 PMC7202438

[44] Abi Abdallah DS, Egan CE, Butcher BA, Denkers EY 2011 Mouse neutrophils are professional antigen-presenting cells programmed to instruct Th1 and Th17 T-cell differentiation. Int Immunol 23:317–326. doi:10.1093/intimm/dxr007. 21422151 PMC3082529

[45] Tsui PT, Kwok ML, Yuen H, Lai ST 2003 Severe acute respiratory syndrome: clinical outcome and prognostic correlates. Emerg Infect Dis 9:1064–1069. doi:10.3201/eid0909.030362. 14519241 PMC3016795

[46] Haick AK, Rzepka JP, Brandon E, Balemba OB, Miura TA 2014 Neutrophils are needed for an effective immune response against pulmonary rat coronavirus infection, but also contribute to pathology. J Gen Virol 95:578–590. doi:10.1099/vir.0.061986-0. 24323639 PMC4093780

[47] Evans SS, Repasky EA, Fisher DT 2015 Fever and the thermal regulation of immunity: the immune system feels the heat. Nat Rev Immunol 15:335–349. doi:10.1038/nri3843. 25976513 PMC4786079

[48] Gorski JP, Hugli TE, Muller-Eberhard HJ 1979 C4a: the third anaphylatoxin of the human complement system. Proc Natl Acad Sci U S A 76:5299–5302. doi:10.1073/pnas.76.10.5299. 291947 PMC413129

[49] Guo RF, Ward PA 2005 Role of C5a in inflammatory responses. Annu Rev Immunol 23:821–852. doi:10.1146/annurev.immunol.23.021704.115835. 15771587

[50] Peng Q, Li K, Sacks SH, Zhou W 2009 The role of anaphylatoxins C3a and C5a in regulating innate and adaptive immune responses. Inflamm Allergy Drug Targets 8:236–246. doi:10.2174/187152809788681038. 19601884

[51] Hu Y, Jin Y, Han D, Zhang G, Cao S, Xie J, Xue J, Li Y, Meng D, Fan X, Sun LQ, Wang M 2012 Mast cell-induced lung injury in mice infected with H5N1 influenza virus. J Virol 86:3347–3356. doi:10.1128/JVI.06053-11. 22238293 PMC3302317

[52] Ram S, Lewis LA, Rice PA 2010 Infections of people with complement deficiencies and patients who have undergone splenectomy. Clin Microbiol Rev 23:740–780. doi:10.1128/CMR.00048-09. 20930072 PMC2952982

[53] Gralinski LE, Menachery VD, Morgan AP, Totura AL, Beall A, Kocher J, Plante J, Harrison-Shostak DC, Schafer A, Pardo-Manuel de Villena F, Ferris MT, Baric RS 2017 Allelic variation in the Toll-like receptor adaptor protein Ticam2 contributes to SARS-coronavirus pathogenesis in mice. G3 (Bethesda) 7:1653–1663. doi:10.1534/g3.117.041434. 28592648 PMC5473747

[54] Totura AL, Whitmore A, Agnihothram S, Schafer A, Katze MG, Heise MT, Baric RS 2015 Toll-like receptor 3 signaling via TRIF contributes to a protective innate immune response to severe acute respiratory syndrome coronavirus infection. mBio 6:e00638-15. doi:10.1128/mBio.00638-15. 26015500 PMC4447251

[55] Poltorak A, He X, Smirnova I, Liu MY, Van Huffel C, Du X, Birdwell D, Alejos E, Silva M, Galanos C, Freudenberg M, Ricciardi-Castagnoli P, Layton B, Beutler B 1998 Defective LPS signaling in C3H/HeJ and C57BL/10ScCr mice: mutations in Tlr4 gene. Science 282:2085–2088. doi:10.1126/science.282.5396.2085. 9851930

[56] Hoshino K, Takeuchi O, Kawai T, Sanjo H, Ogawa T, Takeda Y, Takeda K, Akira S 1999 Cutting edge: Toll-like receptor 4 (TLR4)-deficient mice are hyporesponsive to lipopolysaccharide: evidence for TLR4 as the lps gene product. J Immunol 162:3749–3752. 10201887

[57] Gralinski LE, Ferris MT, Aylor DL, Whitmore AC, Green R, Frieman MB, Deming D, Menachery VD, Miller DR, Buus RJ, Bell TA, Churchill GA, Threadgill DW, Katze MG, McMillan L, Valdar W, Heise MT, Pardo-Manuel de Villena F, Baric RS 2015 Genome wide identification of SARS-CoV susceptibility loci using the collaborative cross. PLoS Genet 11:e1005504. doi:10.1371/journal.pgen.1005504. 26452100 PMC4599853

[58] Fett C, DeDiego ML, Regla-Nava JA, Enjuanes L, Perlman S 2013 Complete protection against severe acute respiratory syndrome coronavirus-mediated lethal respiratory disease in aged mice by immunization with a mouse-adapted virus lacking E protein. J Virol 87:6551–6559. doi:10.1128/JVI.00087-13. 23576515 PMC3676143

[59] Franks TJ, Chong PY, Chui P, Galvin JR, Lourens RM, Reid AH, Selbs E, Mcevoy CPL, Hayden CDL, Fukuoka J, Taubenberger JK, Travis WD 2003 Lung pathology of severe acute respiratory syndrome (SARS): a study of 8 autopsy cases from Singapore. Hum Pathol 34:743–748. doi:10.1016/S0046-8177(03)00367-8. 14506633 PMC7119137

[60] Naito AT, Sumida T, Nomura S, Liu ML, Higo T, Nakagawa A, Okada K, Sakai T, Hashimoto A, Hara Y, Shimizu I, Zhu W, Toko H, Katada A, Akazawa H, Oka T, Lee JK, Minamino T, Nagai T, Walsh K, Kikuchi A, Matsumoto M, Botto M, Shiojima I, Komuro I 2012 Complement C1q activates canonical wnt signaling and promotes aging-related phenotypes. Cell 149:1298–1313. doi:10.1016/j.cell.2012.03.047. 22682250 PMC3529917

[61] McGeer EG, Klegeris A, McGeer PL 2005 Inflammation, the complement system and the diseases of aging. Neurobiol Aging 26:94–97. doi:10.1016/j.neurobiolaging.2005.08.008. 16198446

[62] Hammerschmidt DE, Weaver LJ, Hudson LD, Craddock PR, Jacob HS 1980 Association of complement activation and elevated plasma-C5a with adult respiratory distress syndrome. Pathophysiological relevance and possible prognostic value. Lancet i:947–949. 10.1016/s0140-6736(80)91403-8 6103300

[63] Hosea S, Brown E, Hammer C, Frank M 1980 Role of complement activation in a model of adult respiratory distress syndrome. J Clin Invest 66:375–382. doi:10.1172/JCI109866. 7400321 PMC371720

[64] Sheahan TP, Sims AC, Graham RL, Menachery VD, Gralinski LE, Case JB, Leist SR, Pyrc K, Feng JY, Trantcheva I, Bannister R, Park Y, Babusis D, Clarke MO, Mackman RL, Spahn JE, Palmiotti CA, Siegel D, Ray AS, Cihlar T, Jordan R, Denison MR, Baric RS 2017 Broad-spectrum antiviral GS-5734 inhibits both epidemic and zoonotic coronaviruses. Sci Transl Med 9:eaal3653. doi:10.1126/scitranslmed.aal3653. 28659436 PMC5567817

[65] Roberts A, Deming D, Paddock C, Cheng A, Yount B, Vogel L, Herman BD, Sheahan T, Heise M, Genrich GL, Zaki SR, Baric R, Subbarao K 2007 A mouse adapted SARS coronavirus causes disease and mortality in BALB/c mice. PLoS Pathog 3:e5. doi:10.1371/journal.ppat.0030005. 17222058 PMC1769406

[66] Yount B, Curtis K, Fritz E, Hensley L, Jahrling P, Prentice E, Denison M, Geisbert T, Baric R 2003 Reverse genetics with a full length infectious cDNA of the severe acute respiratory syndrome coronavirus. Proc Natl Acad Sci U S A 100:12995–13000. doi:10.1073/pnas.1735582100. 14569023 PMC240733

[67] Sheahan T, Rockx B, Donaldson E, Sims A, Pickles R, Corti D, Baric R 2008 Mechanisms of zoonotic severe acute respiratory syndrome coronavirus host range expansion in human airway epithelium. J Virol 82:2274–2285. doi:10.1128/JVI.02041-07. 18094188 PMC2258931

[68] Misharin AV, Morales-Nebreda L, Mutlu GM, Budinger GR, Perlman H 2013 Flow cytometric analysis of macrophages and dendritic cell subsets in the mouse lung. Am J Respir Cell Mol Biol 49:503–510. doi:10.1165/rcmb.2013-0086MA. 23672262 PMC3824047

