# Supplementary figures and images for: Complement Activation Contributes to Severe Acute Respiratory Syndrome Coronavirus Pathogenesis
Source: mBio. 2018 Oct 9;9(5):e01753-18. doi: 10.1128/mBio.01753-18 (PMC6178621; doi:10.1128/mBio.01753-18)

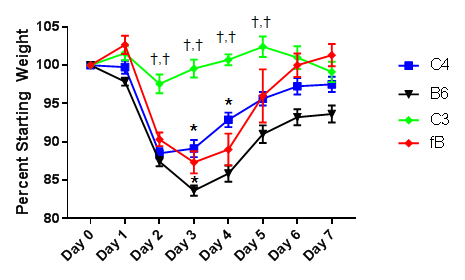

Supplement: FIG S1 [file mbo005184095sf1.tif]

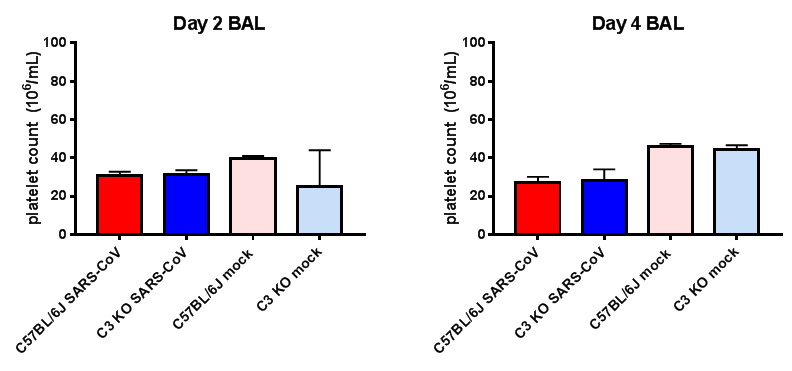

Supplement: FIG S2 [file mbo005184095sf2.tif]
