# Supplementary material for: Complement Activation Contributes to Severe Acute Respiratory Syndrome Coronavirus Pathogenesis
Source: mBio. 2018 Oct 9;9(5):e01753-18. doi: 10.1128/mBio.01753-18 (PMC6178621; doi:10.1128/mBio.01753-18)
Supplement: TABLE S1 [file mbo005184095st1.docx]

|  |  | **Denudation** | **Debris** | **Infl.** | **Perivascular Cuffing** | **Edema** | **Interstitial Septum Thickening** | **Airspace Infl.** | **Hyaline Membrane** | **Exudates** | **Eos** |
| --- | --- | --- | --- | --- | --- | --- | --- | --- | --- | --- | --- |
| Day 4 | B6 inf | 0.5 | 0.88 | 0.94 | 1.06 | 0.31 | 1.06 | 1.31 | 0 | 0.69 | 0.88 |
|  | C3 inf | 0.5 | 0.56 | 0.5 | 0.63 | 0.13 | 0.38 | 0.25 | 0 | 0.06 | 0.25 |
|  | B6 mock | 0 | 0 | 0 | 0 | 0 | 0 | 0 | 0 | 0 | 0 |
|  | C3 mock | 0.25 | 1 | 0 | 0.25 | 0 | 0 | 0 | 0 | 0 | 0 |
|  |  |  |  |  |  |  |  |  |  |  |  |
| Day 2 | B6 inf | 0.75 | 1.67 | 0.92 | 0.92 | 0.25 | 1 | 0.75 | 0 | 0.42 | 0.83 |
|  | C3 inf | 0.88 | 1.88 | 1 | 1.13 | 0.63 | 1.25 | 1.25 | 0 | 0.5 | 1.25 |
|  | B6 mock | 0 | 0.25 | 0.13 | 0.13 | 0 | 0.38 | 0.13 | 0 | 0 | 0.25 |
|  | C3 mock | 0 | 0.38 | 0.25 | 0.25 | 0 | 0.25 | 0 | 0 | 0.25 | 0 |
